# Artificial Dilatation of the Os Uteri

**Published:** 1845-04

**Authors:** John Breen

**Affiliations:** Formerly Assistant Physician to the Lying-in Hospital, Dublin


*Artificial Dilatation of the Os Uteri.* By John Breen, M.D., formerly Assistant Physician to the Lying-in Hospital, Dublin. The late Dr. Hamilton, it is well known, stated that by securing the completion of the first stage of labour within twelve or fourteen hours, no patient under his charge for the thirty-five years antecedent to his publication, had been above twenty-four hours in labour, and, except in cases of disproportion, none so long. We are convinced that the practice of Dr. Hamilton, supported, as it now is, by the experience of many eminent and practical men, has been too long neglected; and we are happy in having the opportunity of publishing the experience of Dr. Breen on this subject. We think, in cases of tedious labour, where the great impediment to the completion of the process is the undilated state of the os uteri, that cautious and very gradual artificial dilatation is perfectly safe and will save the patient many hours of unnecessary pain. Most of our readers will be aware of the mode adopted by Dr. Hamilton and Mr. Burns for this purpose. Dr. Breen's method is as follows. He says:

I think artificial dilatation should not be attempted for at least ten hours after the real commencement of labour, no matter what the duration of spurious labour may have previously been. When this measure is called for the membranes will be usually found ruptured, but not universally so. Two fingers are to be introduced, either between the os uteri and unruptured membranes, or, if ruptured, between the os and presenting part of the child. The fingers are to be used either for the purpose of dilatation or as a wedge, to resist the coming down of a flap of the cervix of that viscus. Above all things it is to be particularly kept in mind that the fingers are to be quiescent except during a pain, and to be used only during the most severe portion of this action. By thus proceeding no additional suffering will be caused to the patient. About two hours will usually be found sufficient to obliterate the undilated cervix, but the attendant need not be anxious to complete this measure within so short a time. He may occasionally cease from his trials and watch what nature may be capable of effecting. By following these rules we avoid the risk of encountering the tendency to inflammatory action, which is always to be dreaded when severe labour is complicated with a long duration of that process. When first I read Mr. Burns's opinions on this subject, now many years back, I was surprised that he did not recommend artificial dilatation to be preceded by blood-letting. This measure, though not contra-indicated, will generally be found unnecessary. In thus bringing the presentation to act on the perineum within the first twenty hours of labour, or generally a shorter period, we secure such a portion of uterine action to be directed to the dilatation of this part, as, if it does not absolutely terminate the expulsion of the child before danger impends, from the protection of parturition, will render instruments compatible with the mother's and child's safety applicable. Sufficient materials exist to prove that the following of the practice advocated by Professor Hamilton, and supported in this essay, will not render obstetric practice either too artificial or meddlesome. For though nature has carefully provided a set of organs for the continuance of the human species, and for the safety of this process, yet, in a certain number of cases, all agree that artificial interference of some kind becomes absolutely necessary. Though it be true that the decree has gone forth that the woman in sorrow shall bring forth children, this is no reason why the obstetrican should not resort to the aid of art, guided by science and experience, to alleviate the suffering of his patient.

I acknowledge I am sanguine enough to expect that the practice of widwifery is capable of being so far improved that the perforator will not be used in cases uncomplicated with obstetric casualties, such as deficiency of space in the pelvis, convulsions, &c., or in cases where the prolapsus of the umbilical cord, joined with a cessation of its pulsation and other certain indications of the child's death, remove all moral objection to this always uncomfortable measure to the conscientious practitioner. In fine, I would appeal to the best informed obstetrician if ever, in the most remote vista of future improvement, he perceives any probability of banishing from practice what, in a former part of this paper, I have called the *opprobrium obstetricorum*, except through the adoption of Mr. Burns's and Professor Hamilton's mode of acting in labour likely to be tedious. No person can doubt that I have added to the Edinburgh professor's authority additional facts bearing on the subject, and supporting the safety of the practice. Let no practitioner, on merely speculative grounds, reject a mode of conducting a labour which mitigates the sufferings of his patient, and much lessens the danger of inflammatory action or debility succeeding parturition—the first rendering the use of every instrumental aid dangerous, and the second very generally proving fatal.—*Lancet.*

